# Isolation and identification of lactic acid bacteria with phytase activity from sourdough

**DOI:** 10.1002/fsn3.1229

**Published:** 2019-10-21

**Authors:** Matin Mohammadi‐Kouchesfahani, Zohreh Hamidi‐Esfahani, Mohammad Hossein Azizi

**Affiliations:** ^1^ Department of Food Science and Technology Faculty of Agriculture Tarbiat Modares University Tehran Iran

**Keywords:** isolation, lactic acid bacteria, phytase activity, phytic acid, sourdough

## Abstract

Wholemeal bread is strongly recommended due to its nutritional value. However, whole‐grain foods contain a high level of phytic acid, an antinutritional factor that decreases the mineral bioavailability. The objective of this study was isolation and identification of lactic acid bacteria with phytase activity to find a suitable starter for bread‐making. Wheat–legume sourdoughs were prepared by the back‐slopping procedure. Lactic acid bacteria were isolated from the sourdough of wheat flour–mung bean, and their phytase activity was tested in the solid and liquid media. Out of the nine phytase‐active isolates in the solid medium, only three isolates produced extracellular phytase in the liquid medium with activity ranging from 16.3 to 53.2 (U/ml). These isolates belonged to species *Weissella confusa mk.zh95* and *Pediococcus pentosaceus*. The highest phytase activity was found for *Weissella confusa mk.zh95*. *Weissella confusa mk.zh95* is considered an interesting source of phytase during cereals and legumes fermentation which improves the bioavailability of minerals.

## 1 INTRODUCTION

Nowadays, consumption of wholemeal bread has dramatically increased due to growing awareness of its nutritional values such as dietary fiber, complex carbohydrates, protein, vitamins, and minerals (Adefegha, Olasehinde, & Oboh, 2018). However, a significant amount of phytic acid (myo‐inositol hexakis phosphoric acid) in wholemeal bread interferes with mineral uptake (Fe^+2^, Zn^+2^, Ca^+2^, Mg^+2^, Cu^+2^, and Mn^+2^; Najafi, Rezaei, Safari, & Razavi, 2012; Nuobariene, Hansen, & Arneborg, 2012; Reale, Konietzny, Coppola, Sorrentino, & Greiner, 2007). Furthermore, phytic acid decreases the activity of enzymes such as trypsin, pepsin, α‐amylase, and β‐galactosidase (Anastasio et al., 2010). Phytic acid is the major form of phosphorus in many cereal grains, legumes, oilseeds, and plant seeds, including wheat and mung bean (Fischer, Egli, Aeberli, Hurrell, & Meile, 2014).

Phytic acid degrading enzymes such as phytase are able to break phosphomonoester bonds of phytic acid. Phytase is endogenously found in cereals, legumes, and microorganisms including lactic acid bacteria (LAB; Hammes et al., 2005; Nuobariene et al., 2015; Palacios, Haros, Sanz, & Rosell, 2008). Phytase degrades phytic acid to lower inositol phosphate esters and inorganic phosphate. Only, myo‐inositol hexakis phosphate (IP6) and inositol pentaphosphate (IP5) interfere with the bioavailability of minerals (Garcıa‐Estepa, Guerra‐Hernández, & Garcıa‐Villanova, 1999).

Phytase activity depends on various factors including temperature, pH, proteolysis resistance, and substrate specificity (Anastasio et al., 2010). Recent studies have shown that the addition of sourdough may increase phytate hydrolysis. Sourdough fermentation is a traditional method exerted to enhance nutritional, functional, and process‐ability features of cereals. Moreover, the use of LAB as a starter culture in sourdough mainly improves the nutritional, textural, sensory, and shelf life properties of bakery products (Corona et al., 2016; Katina, Salmenkallio‐Marttila, Partanen, Forssell, & Autio, 2006; Lopez et al., 2003; Torrieri, Pepe, Ventorino, Masi, & Cavella, 2014). Recently, the use of indigenous starter cultures for cereal fermentation and the manufacture of bakery products have received much attention.

Most studies indicated that LAB did not exhibit significant extracellular phytase activity (De Angelis et al., 2003; Fredrikson, Andlid, Haikara, & Sandberg, 2002; Goswami, Bora, Parveen, Boro, & Barooah, 2017; Palacios, Haros, Rosell, & Sanz, 2005; Reale et al., 2007; Zamudio, Gonzalez, & Medina, 2001), whereas only a few studies have reported LAB with extracellular phytase activity (Anastasio et al., 2010; Nuobariene et al., 2015). Intracellular phytase enclosed within the cell does not have access to phytic acid unless the cell breakdown. Therefore, application of LAB with extracellular phytase activity as a starter culture in bread‐making is more efficient than LAB with intracellular phytase activity.

Legumes and wheat bran are rich sources of proteins, dietary fibers, and carbohydrates. Moreover, they contain minerals, essential amino acids, vitamins, and phenolic compounds and decrease the risk of heart disease, diabetes, and some types of cancer (Curiel et al., 2015).

The objective of the current study was to isolate LAB with extracellular phytase activity from different sourdoughs of wheat–legume to identify phytase‐active strains that could be used as a starter culture in bread‐making.

## 2 MATERIALS AND METHODS

### 2.1 Materials

Sodium phytate was purchased from Sigma‐Aldrich. All other chemicals, solvents, and media were purchased from Merck.

### 2.2 Wheat and legume flour

Domestic wheat flour and bran (*Triticum aestivum*) were purchased from Azargandom Company. Red lentil (*Lens culinaris*) and mung bean (*Vigna radiate*) were purchased from a local market and milled by a laboratory mill (Cyclotec 1093, Tecator) after cleaning, washing, and drying. The physicochemical characteristics of bran and flours were determined according to AACC standard methods (AACC, 2002).

### 2.3 Preparation and propagation of sourdoughs

Preliminarily, wheat flour, legume flour, and tap water were mixed to prepare nine different sourdoughs according to Table 1. Dough yields (DY) were 160 (DY = (dough weight × 100)/flour weight). All the doughs were incubated at 30°C for 6 hr. After spontaneous fermentation, the sourdoughs were kept at 10°C for 18 hr. Then, the back‐slopping procedure was carried out by replacing 15% (w/w) of the formulations mentioned above with the previous step sourdough (Rizzello, Calasso, Campanella, Angelis, & Gobbetti, 2014). Ten refreshment steps were performed to obtain mature sourdoughs according to the traditional type I sourdough. This process kept dominating microorganisms metabolically in the active form (Ercolini et al., 2013; Rizzello et al., 2014).

**Table 1:** Dry ingredients (%) used for sourdough making

Sourdough sample^a^	Wheat flour	Wheat bran	Red lentil flour	Mung bean flour
W_60_B_30_L_10_	60	30	10	–
W_60_B_30_M_10_	60	30	–	10
W_60_B_30_L_5_M_5_	60	30	5	5
W_40_B_50_L_10_	40	50	10	–
W_40_B_50_M_10_	40	50	–	10
W_40_B_50_L_5_M_5_	40	50	5	5
W_20_B_70_L_10_	20	70	10	–
W_20_B_70_M_10_	20	70	–	10
W_20_B_70_L_5_M_5_	20	70	5	5

a W = wheat flour, B = wheat bran, L = red lentil flour, and M = mung bean flour. Subscripts are the percentage of compositions.

### 2.4 Determination of pH and total titratable acidity (TTA)

The pH values were measured by a pH meter (CONSORT P207, SCHOTT Belgium). Ten grams of each sample were suspended in 90 ml distilled water and homogenized. Then, the pH value of the supernatant liquid was determined by the pH meter. Subsequently, total titratable acidity (TTA) of the samples was determined by titration with 0.1 mol/L NaOH to a final pH of 8.5 ± 0.1 (Nuobariene, Arneborg, & Hansen, 2014; Nuobariene et al., 2012).

### 2.5 Determination of phytic acid

The phytic acid content was measured according to the method described by Garcıa‐Estepa et al. (1999). The phytic acid of the samples was extracted by shaking 40.0 ml of the extraction solution (10 g/100 g sodium sulfate in 0.4 mol/L hydrochloric acid) for 3 hr at room temperature and centrifugation for 30 min at 5,000 *g*. The supernatant (10.0 ml) was pipetted into a 100‐ml centrifuge tube and mixed with 10.0 ml of 0.4 mol/L HCl, 10.0 ml of 0.02 mol/L FeCl_3_ and 10.0 ml of 20% (w/w) sulphosalicylic acid. The tube was shaken and sealed by a rubber cork through which passed a thin glass pipe to prevent evaporation. The tube was incubated in a boiling water bath for 15 min and then cooled down. The suspension was centrifuged at 5,000 *g* for 10 min, filtered, pipetted into a 100‐ml volumetric flask, and diluted. Afterward, 20 ml of the homogeneous solution was removed and the pH was adjusted to 2.5 by adding glycine. Then, the solution was diluted to 200 ml and the volumetric flask was placed in a water bath (70–80°C). The warm solution was titrated with 50 mmol/L ethylenediamine tetraacetic acid (EDTA). The phytic acid content of the samples was determined according to Equations (1), (2), (3), (4).

(1) $$A=V\times C_{1}\times 55.845$$

where *V* is the original FeCl_3_ volume (10.0 ml), *C*_1_ is the original FeCl_3_ concentration (0.02 mol/L), and *A* is the milligram of original Fe.

(2) $$B=C_{2}\times V_{2}\times 10\times 0.1\times 55.845/V_{3}$$

(3) $$M=\left(A-B\right)\times 6\times 660.04/\left(4\times 55.845\right)$$

where *C*_2_ is the EDTA concentration (mol/L), *V*_2_ is the volume of EDTA used in titration (ml), *V*_3_ is the final sample volume (ml), *B* is the milligrams of residual Fe, and *M* is the milligram of phytic acid in the sample.

(4) $$S=\left(M/W\right)\times 100$$

where *W* is the original weight of the sample (g) and *S* is the phytic acid content of the sample (mg/100 g).

### 2.6 Determination of minerals and trace element

Minerals and trace elements in the samples were determined using a multi‐element technique inductively coupled plasma optical emission spectrometry (ICP‐OES) according to method 985.01 from AOAC (AOAC, 2006). The samples (1.0 g) were placed in a furnace at 450°C for 16 hr, whereas ashing temperature was 500°C in the method 985.01 from AOAC. A lower temperature and longer time were used for ashing to reduce the risk of losing minerals due to the formation of volatile substances (Kopec et al., 2011). The ash digestion was carried out using 5 ml concentrated nitric acid on a hot plate. The digested sample was filtered by Whatman filter paper. Finally, the mineral contents of the clear solution were measured by ICP‐OES (Varian Inc. 730‐ES; Altundag & Tuzen, 2011). The calibration solutions were prepared by diluting stock solutions. The five‐point calibration curves were selected, and the concentration of elements in samples was calculated. The detection limits (LOD) of the method were 0.01, 0.2, 0.1, 0.01, and 1.5 μg/L for Ca, Zn, Fe, Mg, and P, respectively. Minerals and trace elements in the samples (Ca, Zn, Fe, Mg, and P) were determined by ICP‐OES using wavelengths 318.12, 206.2, 259.94, 280.27, and 220.35 nm, respectively.

### 2.7 Enumeration of lactic acid bacteria (LAB) and yeasts

Cell densities of the LAB were determined by plating onto de Man, Rogosa and Sharpe agar (MRS agar) media. Incubation was carried out under anaerobic conditions at 30°C for 48 hr. The yeasts were counted on Yeast Extract Glucose Chloramphenicol agar (YGC) at 25°C for 72 hr (Curiel et al., 2015; Nionelli et al., 2014).

### 2.8 Isolation of lactic acid bacteria (LAB)

Five to ten colonies of the presumptive LAB were randomly selected from the plates containing the two highest dilutions. Gram‐positive, catalase‐negative, nonmotile rod, and cocci isolates were recultivated in MRS broth at 30°C for 24 hr. A pure culture of the LAB was obtained by restreaking onto MRS agar (Nionelli et al., 2014).

### 2.9 Screening of lactic acid bacteria for phytase activity in the solid medium

Screening of the LAB for phytase activity was performed according to the methods described by Volfova, Dvorakova, Hanzlikova, and Jandera (1994) and Manini et al. (2016) with some modifications. Briefly, the LAB were cultivated on modified Chalmers broth without neutral red and with 1% of sodium phytate at 30°C for 48 hr. Subsequently, five microliters of microbial suspension were spotted on modified Chalmers agar without CaCO_3_ and with 1% of sodium phytate at 30°C for 48 hr. The plates were examined for clearing zones around the spots. Small red colonies with a light halo were considered as phytase‐active LAB. The plates were flooded with a 2% (w/v) aqueous cobalt chloride solution to avoid false‐positive results due to the acid dissolution of the media. The petri dishes were flooded with a molybdate/vanadate solution to change the color from pink to yellow and increase contrast (Bae, Yanke, Cheng, & Selinger, 1999).

### 2.10 Phytase assay in a liquid medium

Lactic acid bacteria strains with phytase activity in the solid medium were selected for phytase assay. For this, the selected LAB were cultivated in modified Chalmers broth without neutral red and with 1% of sodium phytate at 30°C for 24 hr (Anastasio et al., 2010). Extracellular phytase extracts were prepared according to the method described previously by Nuobariene, Hansen, Jespersen, and Arneborg (2011). The samples were centrifuged at 10,000 *g* for 10 min at 4°C. Supernatants were collected and filtered by 0.2 µm Minisart filters (Nuobariene et al., 2011). Thereafter, the assay was performed by mixing 500 µl of the cell‐free supernatants with 250 µl of 0.2 M sodium acetate buffer (pH 5.15) and 5 mM sodium phytate. The reaction mixtures were incubated at 40°C in a water bath for 45 min. The reaction was stopped by adding 1 ml of 15% (w/v) trichloroacetic acid solution (TCA). Subsequently, 500 µl of the reaction mixture was mixed with 4 ml of color reagent prepared daily (2:1:1 v:v:v acetone, 10 mM ammonium molybdate and 5 N sulfuric acid) and 400 µl of 1 M citric acid. The released inorganic phosphate was determined by a UV‐V spectrophotometer (Agilent Technologies, Cary 60) at 355 nm. A standard curve was prepared with potassium dihydrogen phosphate (KH_2_PO_4_; Heinonen & Lahti, 1981).

One unit of phytase activity (U) was defined as the amount of enzyme required to release 1 µmol of inorganic phosphate per minute from a 5 mM sodium phytate solution at pH 5.15 and a temperature of 40°C.

### 2.11 DNA extraction, genotypic characterization, and identification

The genomic DNA of the isolated LAB with phytase activity was extracted by using a DNA extraction kit (Roche). Afterward, universal primers 27F (5′‐AGAGTTTGATCCTGGCTCAG‐3′) and 1492R (5′‐GGTTACCTTGTTACGACTT‐3′) were used to amplify approximately 1,500 bp of 16SrDNA. The polymerase chain reaction products were verified by agarose gel electrophoresis, purified by using a purification kit (Roche), and sequenced. The sequences were analyzed by the BLAST algorithm, and similarities were searched for within the GenBank available in the NCBI database (Altschul et al., 1997).

### 2.12 Preparation of sourdoughs with phytase‐positive lactic acid bacteria (LAB)

The phytase‐positive LAB were grown in MRS broth at 30°C for 24 hr. The incubated broth cultures were centrifuged at 4,000 *g*, 10 min, 4°C, washed with distilled sterile water, and suspended in sterile tap water to prepare cell suspension (Kajala et al., 2016). Then, sourdoughs were made in an IM 5e8 high‐speed mixer (Mecnosud) by mixing wheat flour (20%), wheat bran (70%), mung bean flour (10% w/w), and tap water using the cell suspension as a starter culture. The final concentration of the LAB in the doughs was 10^7^ CFU/g. Subsequently, the doughs were covered with an aluminum foil and incubated at 30°C for 16 hr. The control was prepared under the same conditions without the starter culture (phytase‐positive LAB) to distinguish the phytase activity of the sourdoughs related to endogenous phytase or microbial phytase. Finally, the pH, TTA, and phytase activity of the sourdoughs were measured.

### 2.13 Statistical analysis

Data analysis was performed by one‐way analysis of variance (ANOVA) with IBM SPSS Statistics 20. An LSD comparison was also performed to determine the significant difference among the means (*p* < .05). Results obtained from three repetitions of assays were given as mean ± standard deviation (*SD*).

## 3 RESULTS AND DISCUSSION

### 3.1 Characteristics of flours and bran

The physicochemical characteristics of the flours and bran used in this study are shown in Table 2. The protein content of the samples ranged from 10.15% to 26.53%, and red lentil showed the highest value. The protein content of the mung bean (25.77%) and red lentil (26.53%) was similar to what was reported by other authors. Ebert, Chang, Yan, and Yang (2017) found an equivalent value for the protein of mung bean (25.33%–27.54%). Moreover, the protein content of red lentil reported by Gharibzahedi, Mousavi, Jafari, and Faraji (2012) ranged between 22.5% and 31.17%. The wheat bran contained a considerably higher mineral (Iron, Zinc, Calcium, and Magnesium) compared with the other grains. The highest phytase activity was found in the mung bean flour (779.7 U/ml), and the lower value of phytase activity was observed in the wheat bran (567.5 U/ml). The phytic acid content of the mung bean was 687.3 mg/100 g, whereas the value for the wheat bran was 1,051.6 ± 28.4 mg/100 g. These results are in agreement with the finding of other investigators (Dhole & Reddy, 2015). The differences in the content of nutritional elements can be attributed to genetic variations and environmental conditions. Furthermore, the phytic acid content depends on the yield and extraction rate of flour and extraction method (Garcıa‐Estepa et al., 1999).

**Table 2:** The proximate composition of the flours and bran used for sourdoughs

	Wheat flour	Wheat bran	Mung bean flour	Red lentil flour
Moisture (%)	11.00 ± 0.1	8.29 ± 0.02	8.68 ± 0.04	8.14 ± 0.08
Protein (*N* × 5.7) (%) based on dry matter	10.15 ± 0.08	16.53 ± 0.08	25.77 ± 0.09	26.53 ± 0.09
Ash (%) based on dry matter	0.497 ± 0.005	4.450 ± 0.007	3.865 ± 0.008	2.227 ± 0.004
Phytic acid (mg/100 g) based on dry matter	51.2 ± 0.6	1,051.6 ± 28.4	687.3 ± 6.5	487.0 ± 20.4
Iron (mg/100 g)	1.09 ± 0.18	11.79 ± 0.34	4.46 ± 0.19	5.04 ± 0.18
Zinc (mg/100 g)	0.48 ± 0.02	7.38 ± 0.04	1.99 ± 0.11	2.09 ± 0.02
Calcium (mg/100 g)	13.48 ± 0.04	89.36 ± 0.09	87.82 ± 6.23	34.96 ± 1.61
Magnesium (mg/100 g)	13.38 ± 0.01	311.65 ± 7.93	127.83 ± 7.95	52.36 ± 0.27
Phosphorous (mg/100 g)	76.28 ± 1.02	920.72 ± 4.35	413.06 ± 19.04	265.46 ± 46
Phytase activity (U/ml)	111.5 ± 4.9	567.5 ± 1.3	779.7 ± 3.4	465.6 ± 1.0

Note

Values are mean ± standard deviation of three replicated assays.

### 3.2 Properties of sourdoughs after ten refreshment steps

The acidification properties of the mature sourdoughs are reported in Table 3. The pH was ranged from 3.14 to 3.97, and TTA value was ranged 10.7–16.9. The sourdough W_20_B_70_L_10_ (including wheat flour 20%, wheat bran 70%, and red lentil flour 10%) showed the highest acidity (low pH, high TTA). The lowest acidity was observed in the sourdough W_60_B_30_L_10_ (including wheat flour 60%, wheat bran 30%, and red lentil flour 10%). A negative correlation was found between TTA and pH (*r* = −.913). Furthermore, a positive correlation was observed between the value of TTA and bran content of the sourdoughs (*r* = .969). The correlation between the value of pH and bran content of the sourdoughs was negative (*r* = −.864). In agreement with this study, Salmenkallio‐Marttila, Katina, and Autio (2001) reported that higher bran content of sourdough caused lower pH and higher TTA values.

**Table 3:** Properties of sourdoughs after the ten refreshment steps

Sourdough sample†	pH	TTA	Phytase activity (U/ml)
W_60_B_30_L_10_	4.0 ± 0.0^e^	10.7 ± 0.3^a^	931.3 ± 7.7^c^
W_60_B_30_M_10_	3.9 ± 0.1^d^	11.3 ± 0.3^a^	1,239.0 ± 5.1^h^
W_60_B_30_L_5_M_5_	4.0 ± 0.1^e^	11.0 ± 0.3^a^	985.4 ± 17.6^d^
W_40_B_50_L_10_	3.4 ± 0.1^b^	13.7 ± 0.3^c^	1,113.9 ± 14.6^f^
W_40_B_50_M_10_	3.4 ± 0.0^b^	14.3 ± 0.3^d^	1,146.0 ± 10.5^g^
W_40_B_50_L_5_M_5_	3.4 ± 0.1^c^	13.1 ± 0.2^b^	1,020.9 ± 5.1^e^
W_20_B_70_L_10_	3.1 ± 0.0^a^	16.9 ± 0.2^f^	731.8 ± 5.1^a^
W_20_B_70_M_10_	3.5 ± 0.1^c^	15.5 ± 0.2^e^	1,523.0 ± 10.1^i^
W_20_B_70_L_5_M_5_	3.3 ± 0.1^e^	15.9 ± 0.2^e^	835.0 ± 7.7^b^

Note

Values are mean ± standard deviation of three replicated assays. ^a–i^Different letters indicate significant differences between the mean values within the column (*p* < .05).

^†^ W = wheat flour, B = wheat bran, L = red lentil flour, and M = mung bean flour. Subscripts are the percentage of compositions.

The phytase activity of the sourdoughs is presented in Table 3. The sourdough W_20_B_70_M_10_ (including wheat flour 20%, wheat bran 70%, and mung bean flour 10%) showed the highest phytase activity (1,523.0 U/ml) and was used for further tests. The increase in phytase activity could likely be related to the high enzymatic activity of the mung bean (Table 2). As mentioned in Table 3, the sourdoughs W_60_B_30_M_10_, W_40_B_50_M_10_, and W_20_B_70_M_10_ showed the highest phytase activity probably due to the higher content of mung bean presented in these samples (10% w/w). In contrast to the results of this study, Steiner et al. believed that legume phytase was not important to improve the availability of plant phosphorus for pigs and poultry. It should be noted that the phytase activity of field beans, peas, and lupins was investigated by them. Harvest year and cultivar affect the amount of total phosphorus and phytic acid. Moreover, large differences have been observed in phytase activity depending on genetics and environmental factors and also the methods used for phytase assay (Steiner, Mosenthin, Zimmermann, Greiner, & Roth, 2007).

In the mature sourdough W_20_B_70_M_10_, the cell counts of the LAB were 3 × 10^8^ log CFU/g and the cell density of the yeast was 2 × 10^6^ log CFU/g. In mature sourdoughs, the number of LAB should be >10^8^ CFU/g and the usual LAB: yeast ratio is 100:1 (Ercolini et al., 2013; Gobbetti, 1998). Eleven isolates were preselected based on the tests performed to detect LAB from the sourdough W_20_B_70_M_10_ that exhibited the highest phytase activity and was used for further tests.

### 3.3 Phytase‐positive lactic acid bacteria (LAB) in a solid medium

**Figure 1:**
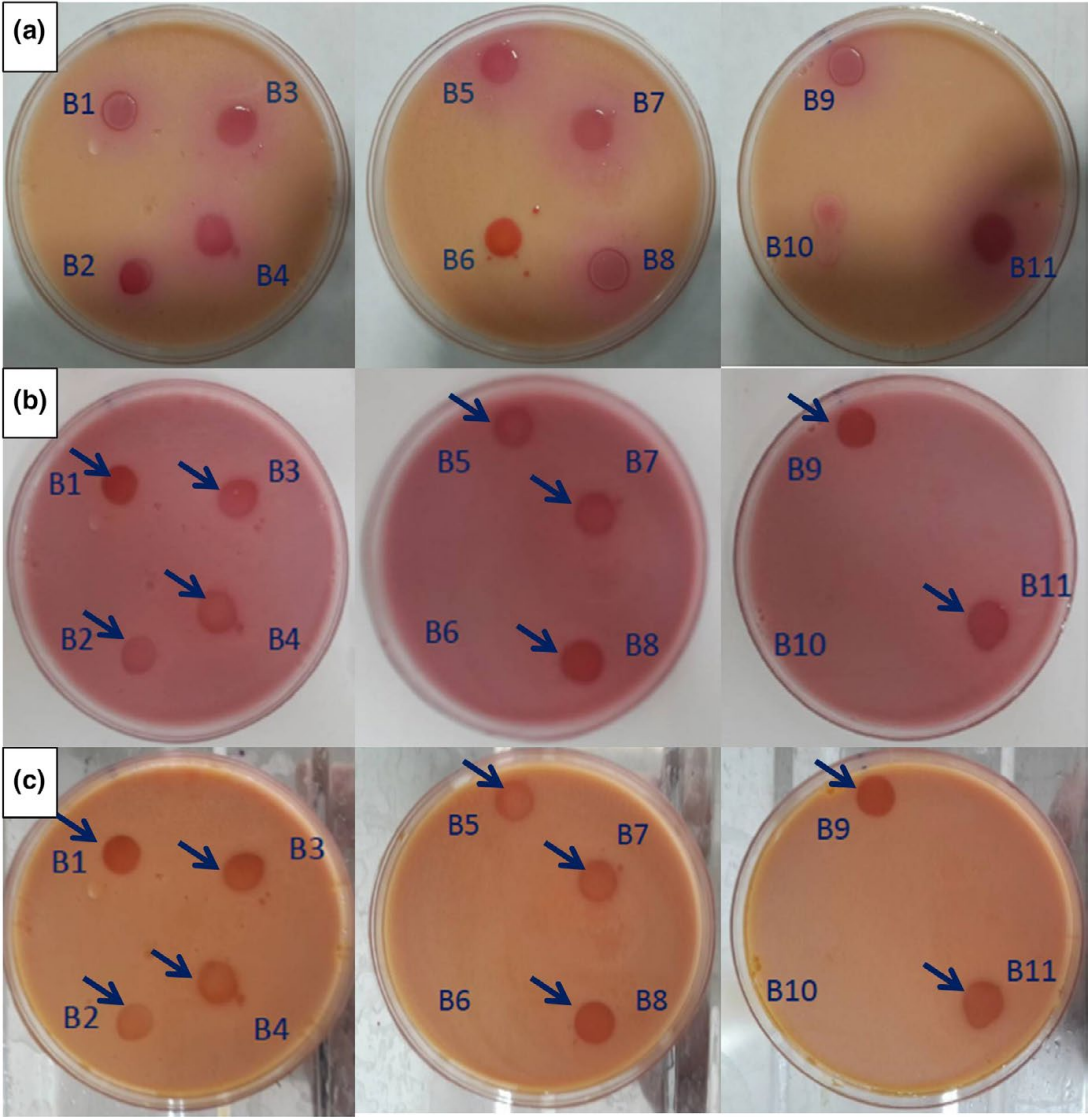
The phytase activity (clearing zone) of the LAB on the modified Chalmers medium (a), elimination of false‐positive results by aqueous cobalt chloride (b), increasing contrast by counterstaining with the molybdate/vanadate solution (c). The arrows indicate phytase‐positive LAB

The dissolution of the precipitated sodium phytate of the modified Chalmers medium can be an appropriate indicator for detecting microbial phytase activity (Bae et al., 1999). All the eleven LAB isolates were tested on the modified Chalmers medium. Nine isolates displayed clearing zones around the colonies ranging from 14 to 20 mm in diameter (Figure 1). False‐positive results caused by the disappearance of the precipitated sodium phytate due to microbial acid production and reduction of pH were eliminated by flooding in a 2% (w/v) cobalt chloride solution. Although the precise mechanism of counterstaining treatment (flooding in the cobalt chloride solution) of media and elimination of false‐positive results is not clearly detected, the binding capacity of phytate with cobalt and metals depends on the pH value. The ability of cobalt chloride for complex forming is less sensitive to pH reduction than other salts. Therefore, cobalt chloride can be an appropriate salt to eliminate false‐positive results (Bae et al., 1999). In our study, flooding in 2% (w/v) aqueous cobalt chloride indicated that the B6 and B10 isolates were phytase‐negative and clearing zones around these colonies resulted in acid production. In agreement with our results, Pepe et al. (2004) reported that six *Lactobacillus plantarum* strains showed phytase activity on modified Chalmers agar by producing clearing haloes around colonies. Furthermore, 8 out of 13 LAB strains were tested on solid media were phytase‐positive (Manini et al., 2016). Numerous phytase‐positive LAB strains were also observed on modified Chalmers agar by Anastasio et al. (2010).

### 3.4 Phytase assay in a liquid medium

The phytase‐active isolates (nine isolates) were selected from the solid medium and tested in a liquid medium. Bae et al. (1999) reported that the phytase activity of 80% of phytase‐positive isolates on solid media could not be confirmed in liquid media unless the counterstaining methods were used for elimination of false‐positive results caused by acid‐producing colonies. The counterstaining method was used in this study; nevertheless, all of the phytase‐active isolates on the modified Chalmers agar did not show phytase activity when tested in the modified Chalmers broth. Similar results were observed by Anastasio et al. (2010). Although the screening method on solid media may be a rapid and cheap method for the detection of phytase activity of a large number of microbial isolates in a complex ecosystem, it is necessary to confirm with phytase assay in liquid media. In this study, three isolates showed phytase activity ranging from 16.3 to 53.2 (U/ml) in liquid media. The highest phytase activity was found for B7 (*Weissella confusa mk.zh95*; Table 4). The previous studies also found phytase‐active LAB strains such as *Lactobacillus acidophilus* 16A, *Lactobacillus brevis* 14G, *Lactobacillus fermentum* 6E, *Lactobacillus lactis* 11M, *Lactobacillus panis* GH1, *Lactobacillus plantarum* DC40, H3, and H5, *Lactobacillus reuteri* 2.3, 8.1, and L‐M15, *Lactobacillus sanfranciscensis* CB1 and 22E, *Leuconostoc citreum* 23B, *Pediococcus pentosaceus* 1.2, and *Weissella confuse* 14A (Anastasio et al., 2010; De Angelis et al., 2003; Nuobariene et al., 2015; Palacios et al., 2008).

**Table 4:** Identification of LAB by partial sequencing of the 16S ribosomal RNA gene

Isolate	Phytase activity (U/ml)^a^	Similarity to GenBank sequence (%)	Closest related LAB	Accession number
B3 (*mk.zh95*)	23.4	98	*Pediococcus pentosaceus*	MG966183
B7 (*mk.zh95*)	53.2	99	*Weissella confusa mk.zh95*	LT715987
B11 (*mk.zh96*)	16.3	93	*Pediococcus pentosaceus*	MG890347

a Values are the mean of three replicated measurements.

Based on the partial sequence of the 16S rRNA gene, the phytase‐active LAB belonged to the species *Weissella confusa mk.zh95* (B7) and *Pediococcus pentosaceus* (B3 and B11). The nucleotide archive accession number of the phytase‐active species is shown in Table 4.

Phytase is important for phytate dephosphorylation and increased mineral bioavailability. Therefore, the use of phytase‐active LAB strains during sourdough fermentation is of interest. Most studies have reported LAB strains with intracellular phytase activity, but LAB strains with extracellular phytase activity have been rarely reported. For example, Reale et al. (2007) indicated that none of the 50 strains of tested LAB produced extracellular phytase in MRS, SDB medium, and phosphate‐reduced media, and also, intracellular phytase activity was ranged between 0.3 and 5.7 mU/ml. Moreover, De Angelis et al. (2003) investigated 13 strains of sourdough LAB and found that intracellular phytase‐active LAB with the highest level of activity belonged to *Lactobacillus sanfranciscensis* CB1 (420.8 U/ml). Since phytate is a common cellular constituent with a significant turnover, intracellular phytase activity might be found in nearly every cell. It is improbable that intracellular phytases are involved in extracellular phytic acid degradation (Reale et al., 2007). Similar to our results, LAB with extracellular phytase activity were found by Anastasio et al. (2010) (*Enterococcus faecium*, *Lactobacillus plantarum T211*, *H10*, *H5*, and *L3* as well as *Leuconostoc gelidum* with activity ranging between 0.53 and 0.74 U/ml). Moreover, *Lactobacillus fermentum*, *Lactobacillus panis*, *Lactobacillus reuteri*, and *Pediococcus pentosaceus* strains exhibited extracellular phytase activity ranging from 3 to 141 U/ml, where *Lactobacillus panis* GH1 showed the highest extracellular phytase activity (Nuobariene et al., 2015). The amount of enzyme activity depends on action conditions and is affected by action condition, measurement methods, and definition of the unit. Therefore, it is difficult to compare the results of various researches.

While several strains of *Pediococcus pentosaceus* are known to be extracellular phytase‐active (Nuobariene et al., 2015), to the best of our knowledge, *Weissella confusa mk.zh95* strains have never been described as an extracellular phytase‐active LAB.

### 3.5 Properties of sourdoughs prepared by phytase‐positive lactic acid bacteria (LAB)

In this study, the effect of phytase‐positive LAB strains was investigated on the pH, TTA, and phytase activity of sourdoughs. Fermentation by phytase‐positive LAB strains caused more acidity compared with the control without the starter culture. The lowest pH (4.0) and highest TTA (14.2) were observed in sourdoughs prepared by *Pediococcus pentosaceus mk.zh95*. Sourdough made by *Weissella confusa mk.zh95* exhibited a mild acidity compared with sourdoughs prepared by *Pediococcus pentosaceus* strains (Table 5).

**Table 5:** Properties of sourdoughs prepared by the phytase‐positive LAB

LAB used as a starter culture	pH	TTA	Phytase activity (U/ml)
Control (uninoculated)	5.0 ± 0.1^c^	7.9 ± 0.2^a^	709.7 ± 12.7^a^
*Pediococcus pentosaceus mk.zh96*	4.1 ± 0.1^ab^	14.0 ± 0.1^c^	805.4 ± 12.4^b^
*Pediococcus pentosaceus mk.zh95*	4.0 ± 0.2^a^	14.2 ± 0.2^c^	889.4 ± 3.3^c^
*Weissella confusa mk.zh95*	4.3 ± 0.2^b^	12.1 ± 0.1^b^	1,058.0 ± 14.1^d^

Note

Values are mean ± standard deviation of three replicated assays. ^a–d^Different letters indicate significant differences between the mean values within the column (*p* < .05).

Significant differences were observed between the sourdoughs in terms of phytase activity (*p* < .05). The control sourdough showed the lowest phytase activity (709.7 U/ml). The highest phytase activity (1,058.0 U/ml) was observed in the sourdough inoculated with *Weissella confusa mk.zh95*. These results were consistent with the data obtained in the phytase assay of LAB (Table 4); for example, sourdough prepared by the highest phytase‐active LAB (*Weissella confusa mk.zh95*) exhibited the highest phytase activity. It seems that phytase activity of sourdough depends on phytase activity of selected LAB strains. In agreement of our result, several other studies have also shown the phytase activity was strain‐dependent (Anastasio et al., 2010; Nuobariene et al., 2015). However, phytase production by LAB strains is one of the controversial issues in the scientific communities. Some scientific data expressed phytase produced by LAB involved in phytic acid degradation, whereas some authors believed that phytic acid degradation during fermentation was independent of the LAB strain and could be due to activation of endogenous cereal phytase as a result of pH reduction. Phytic acid degradation is pH‐dependent, and optimum pH for endogenous cereal phytase is reported between 5 and 5.5 (Bartnik & Szafranska, 1987; Reale et al., 2007). In our study, the control sourdough which had a pH value of 5 exhibited the lowest phytase activity, although according to what they have expressed it was expected to show the highest phytase activity. Moreover, although similar pH and TTA values were obtained for sourdoughs prepared by *Pediococcus pentosaceus mk.zh95* and *Pediococcus pentosaceus mk.zh96*, the phytase activity of sourdoughs was significantly different. It seems that increasing the phytase activity of sourdoughs cannot be only due to pH reduction. Enhancing phytase activity might be as a consequence of activation of endogenous cereal phytase and microbial phytase.

## 4 CONCLUSION

In the present study, the three isolates (*Weissella confusa mk.zh 95*, *Pediococcus pentosaceus mk.zh95*, and *Pediococcus pentosaceus mk.zh96*) showed extracellular phytase activity. Previously, *Weissella confusa mk.zh 95* strains were studied to produce intracellular phytase. To the best of our knowledge, *Weissella confusa mk.zh95* strains have never been described as an extracellular phytase producer. Utilization of LAB with extracellular phytase activity as a starter culture in bread‐making is more important than LAB with intracellular phytase activity as extracellular phytase simply has access to phytic acid in comparison with intracellular phytase enclosed within the cell. Furthermore, the sourdough made by *Weissella confusa mk.zh95* had lower acidity in comparison with sourdoughs made by *Pediococcus pentosaceus* strains. Strong acidification decreased bread volume. *Weissella confusa mk.zh95* is considered an interesting source of phytase during sourdough fermentation which can improve the bioavailability of minerals. Further studies are required to determine the effect of various factors (pH, temperature, and inhibitors) on the phytase activity of LAB and kinetic parameters of enzyme and also to use phytase‐positive LAB as a starter culture in whole‐bread‐ making.

## CONFLICT OF INTEREST

The authors declare that they have no conflicts of interest.

## ETHICAL APPROVAL

This investigation did not involve human or animal testing.
